# Supplementary material for: Low precipitation due to climate change consistently reduces multifunctionality of urban grasslands in mesocosms
Source: PLoS One. 2023 Feb 3;18(2):e0275044. doi: 10.1371/journal.pone.0275044 (PMC9897532; doi:10.1371/journal.pone.0275044)
Supplement: S1 File — (DOCX) [file pone.0275044.s001.docx]

**S1 File. Description of study system**

Our study focuses on grasslands implemented as part of urban green infrastructures. In particular, we reproduced grassland establishment in road verges of heavily urbanized temperate areas (e.g., Munich, Germany). These patches of urban road verge grasslands are characterized by challenging environmental conditions, e.g., shallow soil with scarce rooting space (10 – 50 cm; **Fig S1**), high exposure to heat island effects due to the heavily sealed surrounding vehicular traffic, pollutants, and frequent trampling. With increasing calls to improve greening in cities with reduced available space for large green infrastructure interventions, road verges constitute an often-overlooked opportunity to increase city biodiversity values and support urban ecosystem services delivery (Mody et al., 2020). Current research initiatives take place in urban road verges to improve the ecological understanding of their role as stepping stones for the establishment and dispersion of plants and animals (mainly flying insects; Hopwood 2008). Additionally, the potential delivery of urban ecosystem services in road verges is of interest to promote the multifunctionality of urban ecosystems (Phillips et al. 2020).


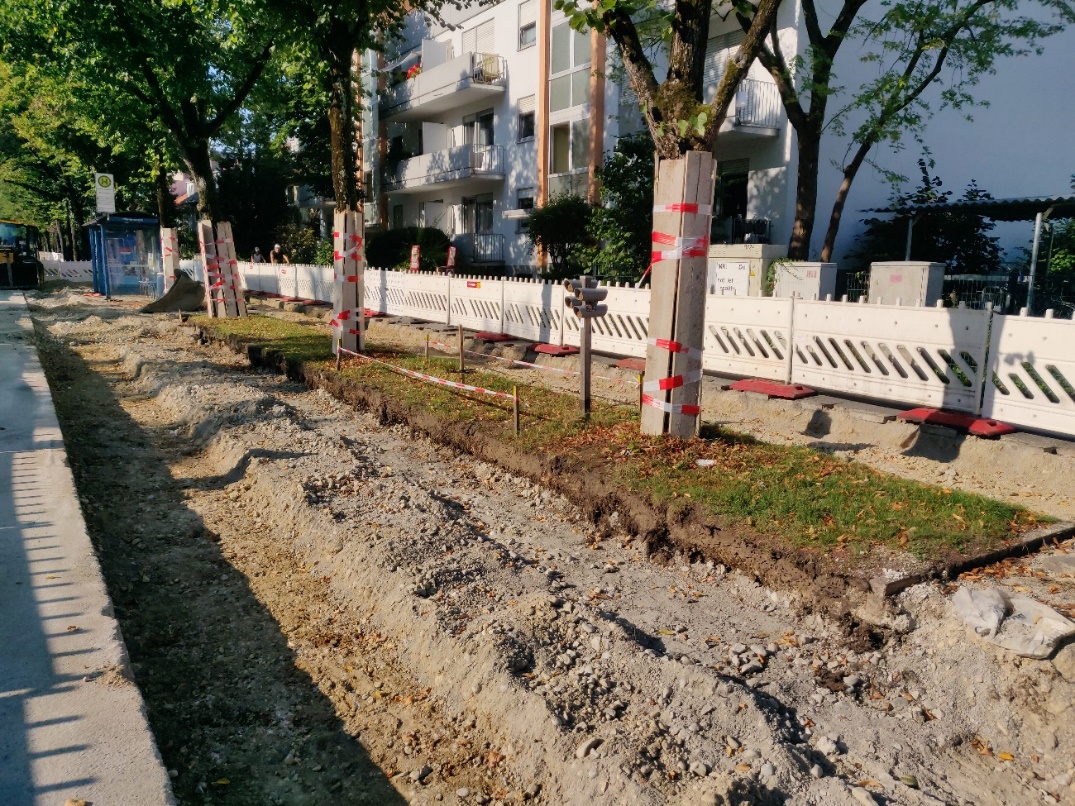


**Fig S1.** Exemplary urban road verge in which restoration measures improve the ecological quality of small patches of grasslands in Munich, Germany. These patches of urban grassland are challenged by restricted space for development (above- and belowground) and heavy concrete sealing of surrounding space. Our experiment simulated these abiotic limitations in mesocosms. Picture: Simon Dietzel.

**Literature cited**

Hopwood, Jennifer L. (2008): The contribution of roadside grassland restorations to native bee conservation. In *Biological Conservation* 141 (10), pp. 2632–2640. DOI: 10.1016/j.biocon.2008.07.026

Mody, Karsten; Lerch, Doris; Müller, Ann-Kathrin; Simons, Nadja K.; Blüthgen, Nico; Harnisch, Matthias (2020): Flower power in the city: Replacing roadside shrubs by wildflower meadows increases insect numbers and reduces maintenance costs. In PloS one 15 (6), e0234327. DOI: 10.1371/journal.pone.0234327

Phillips, Benjamin B.; Bullock, James M.; Osborne, Juliet L.; Gaston, Kevin J. (2020): Ecosystem service provision by road verges. In J Appl Ecol 57 (3), pp. 488–501. DOI: 10.1111/1365-2664.13556
