# Supplementary material for: Low precipitation due to climate change consistently reduces multifunctionality of urban grasslands in mesocosms
Source: PLoS One. 2023 Feb 3;18(2):e0275044. doi: 10.1371/journal.pone.0275044 (PMC9897532; doi:10.1371/journal.pone.0275044)
Supplement: S2 File — (DOCX) [file pone.0275044.s002.docx]

**S2 File. Mesocosm urban grasslands multifunctionalty at 50% threshold level.** Precipitation remains significant for grassland multifunctionality considering either aboveground biomass production (AGB) or floral density (FLO) for the calculation of multifunctionality. Functional composition of grasslands becomes significant for such threshold of multifunctionality either additively or interactively with RCP scenario.

|  | **50% Threshold AGB** | | **50% Threshold FLO** | |
| --- | --- | --- | --- | --- |
|  | **Estimate** | **t stat** | **Estimate** | **t stat** |
| Intercept | 0.50 | 5.20 | 0.39 | 4.15 |
| RCP 8.5 | 0.13 | 1.08 | 0.03 | 0.24 |
| Precipitation_Norm_ | 0.10 | **2.38** | 0.18 | **4.97** |
| F50 | 0.11 | 1.81 | -0.07 | 0.33 |
| F75 | 0.09 | 1.47 | -0.05 | 0.49 |
| F100 | 0.14 | **2.33** | 0.05 | 0.49 |
| RCP 8.5: Precip_Norm_ |  |  |  |  |
| PrecRed:F50 |  |  |  |  |
| PrecRed:F75 |  |  |  |  |
| PrecRed:F100 |  |  |  |  |
| RCP 8.5:F50 |  |  | 0.36 | **3.47** |
| RCP 8.5:F75 |  |  | 0.20 | 1.88 |
| RCP 8.5:F100 |  |  | 0.18 | 1.68 |
| Marg /Cond R^2^ | 0.19/0.44 |  | 0.45/0.64 |  |


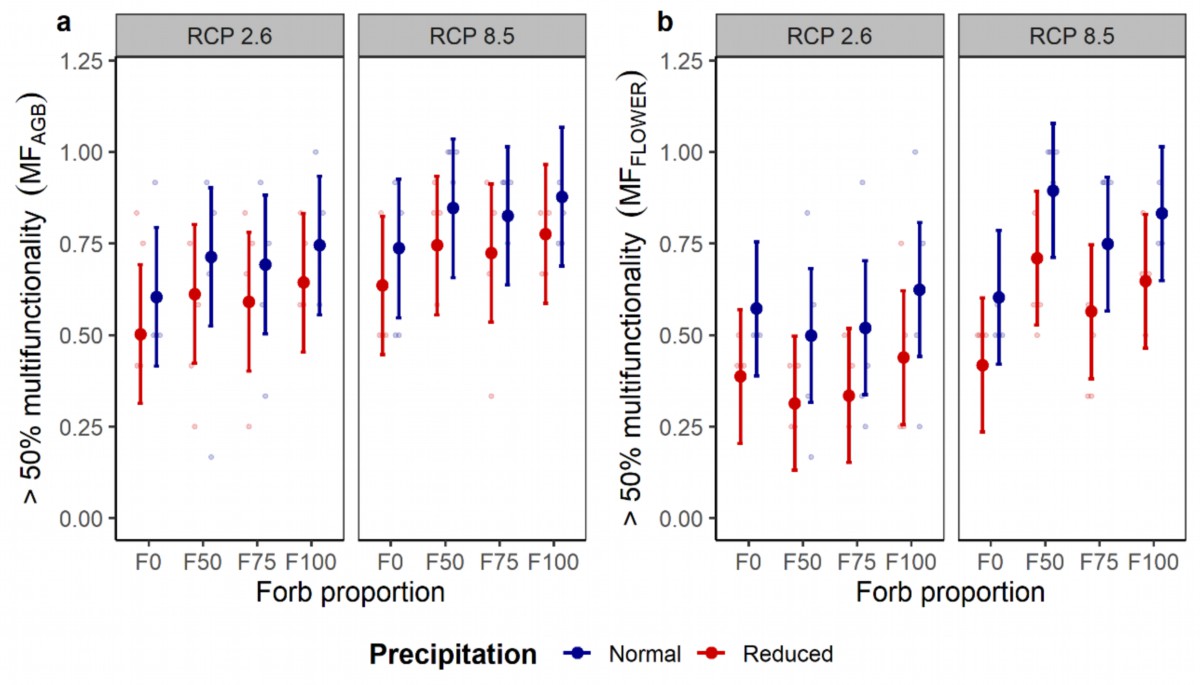


Effects of forb proportion, precipitation, climate change scenario (RCP scenario), and the interaction between RCP scenario and forb proportion on mesocosm grassland multifunctionality at levels equal or exceding 50% of maximum multifunctionality. (a) Multifunctionality index calculated with aboveground biomass and six other indicator variables of grassland functioning, (b) Multifunctionality index calculated with floral density and six other indicator variables of grassland functioning.
