# Supplementary material for: Low precipitation due to climate change consistently reduces multifunctionality of urban grasslands in mesocosms
Source: PLoS One. 2023 Feb 3;18(2):e0275044. doi: 10.1371/journal.pone.0275044 (PMC9897532; doi:10.1371/journal.pone.0275044)
Supplement: S1 Fig — (DOCX) [file pone.0275044.s003.docx]

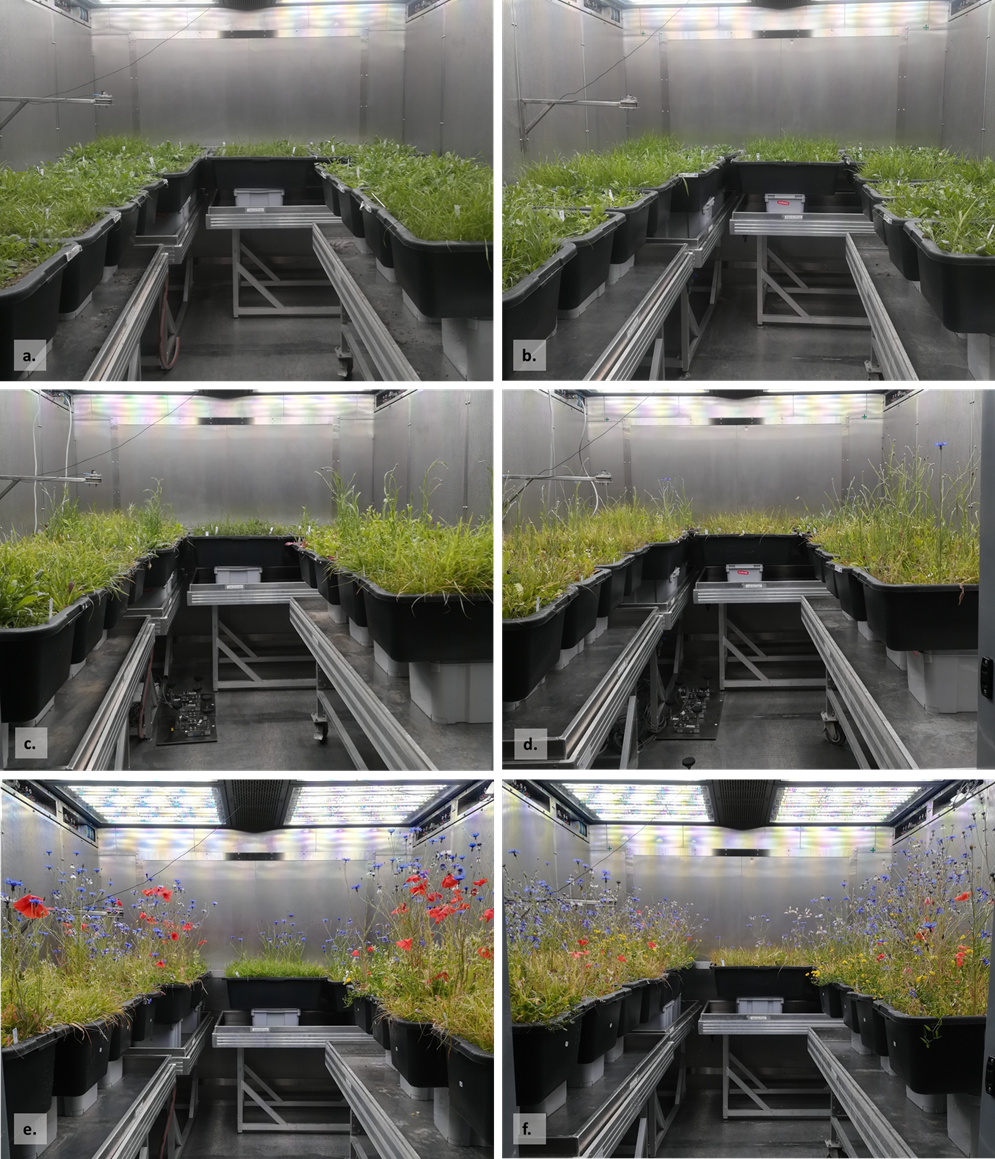


**S1 Fig. *Walk-in* climate chambers of TUM*mesa*** **ecotron facility** **used for the mesocosm experiment with grasslands.** Communities with four different functional compositions were subjected to two climate scenarios (left side, RCP2.6; right side, RCP8.5). Temperature and CO_2_ concentration in the air were manipulated in each chamber, while simulated precipitation (normal vs. reduced) was randomized for each table containing four different experimental communities. Rain simulation was performed with a rainfall-like sprinkler using tap water. Each mesocosm was placed on a grey box to collect excess water. Pictures were taken on a–b) 5^th^ week, c–d) 7^th^ week, and e–f) 10^th^ week of the experiment.
