# Supplementary material for: Low precipitation due to climate change consistently reduces multifunctionality of urban grasslands in mesocosms
Source: PLoS One. 2023 Feb 3;18(2):e0275044. doi: 10.1371/journal.pone.0275044 (PMC9897532; doi:10.1371/journal.pone.0275044)
Supplement: S2 Fig — (DOCX) [file pone.0275044.s004.docx]

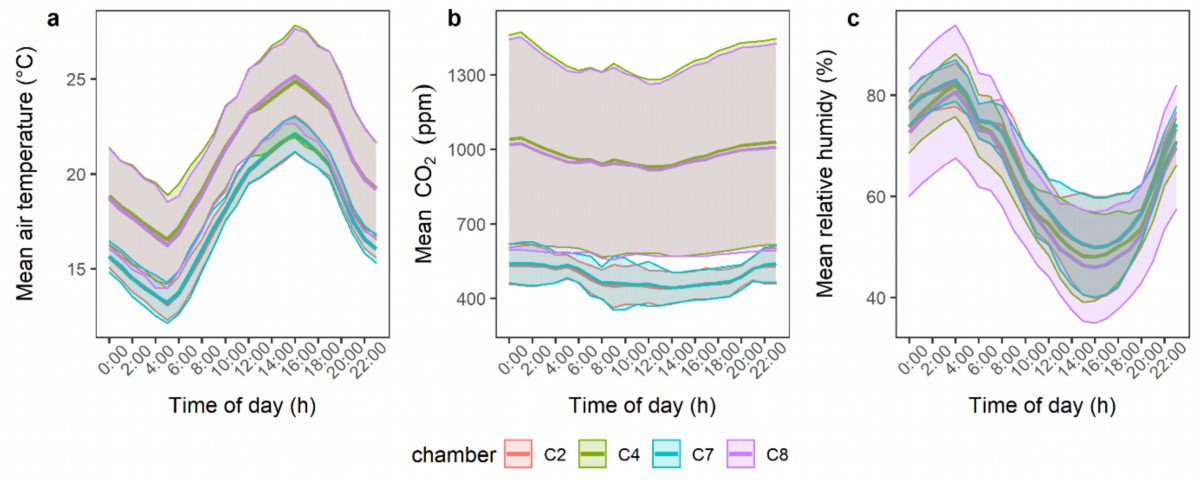


**S2 Fig.** **Realized daily air temperature, CO_2_ concentration, and relative humidity in the climate chambers of the TUMmesa ecotron facility**. Climate change scenarios RCP2.6 (chambers C2 and C7) and RCP8.5 (C4 and C8) were simulated to test the effects of functional composition (proportion forbs vs. grasses), precipitation and RCP scenario on urban grassland functioning. Lines represent mean values over a day, and shaded areas are ±SD.
