# Supplementary material for: Low precipitation due to climate change consistently reduces multifunctionality of urban grasslands in mesocosms
Source: PLoS One. 2023 Feb 3;18(2):e0275044. doi: 10.1371/journal.pone.0275044 (PMC9897532; doi:10.1371/journal.pone.0275044)
Supplement: S3 Fig — (DOCX) [file pone.0275044.s005.docx]

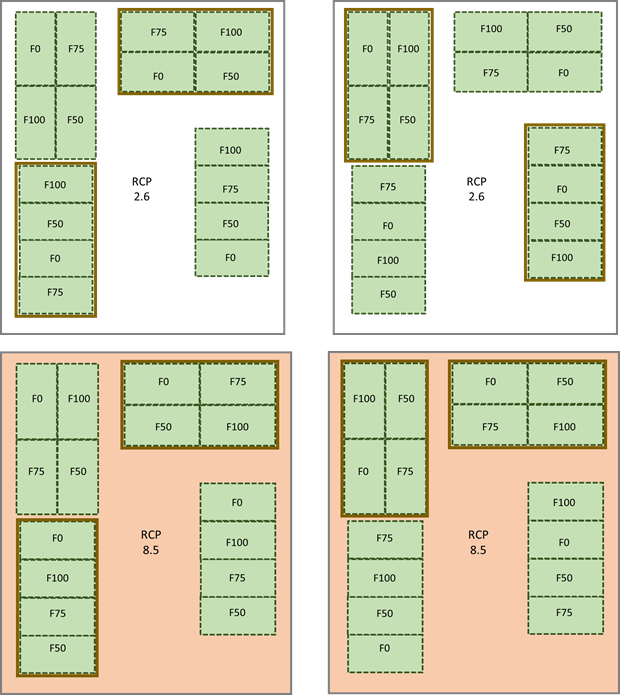


**S3 Fig. Experimental design deployed in the climate chambers of the TUMmesa econtron facility.** We tested the response of mesocosm urban grassland functioning to climate change scenarios (RCP2.6 and 8.5), reduced precipitation (normal and reduced), and four communities with different functional composition, i.e., proportions of forbs vs. grasses (F0, F50, F75, F100). Shown are the climate chambers (large containing squares, light-colored: RCP 2.6; dark: 8.5). Four tables (mid-sized rectangles with solid-green circumvent lines) per chamber and four mesocosms per table (small rectangles with dashed-green circumvent lines) were installed. Precipitation was controlled at table level (brown lines representing tables in which mesocosms received 50% precipitation), while the four community compositions were randomly assigned a location per table. In total, 64 mesocosms were assessed.
