# Supplementary material for: Low precipitation due to climate change consistently reduces multifunctionality of urban grasslands in mesocosms
Source: PLoS One. 2023 Feb 3;18(2):e0275044. doi: 10.1371/journal.pone.0275044 (PMC9897532; doi:10.1371/journal.pone.0275044)
Supplement: S4 Fig — (DOCX) [file pone.0275044.s006.docx]

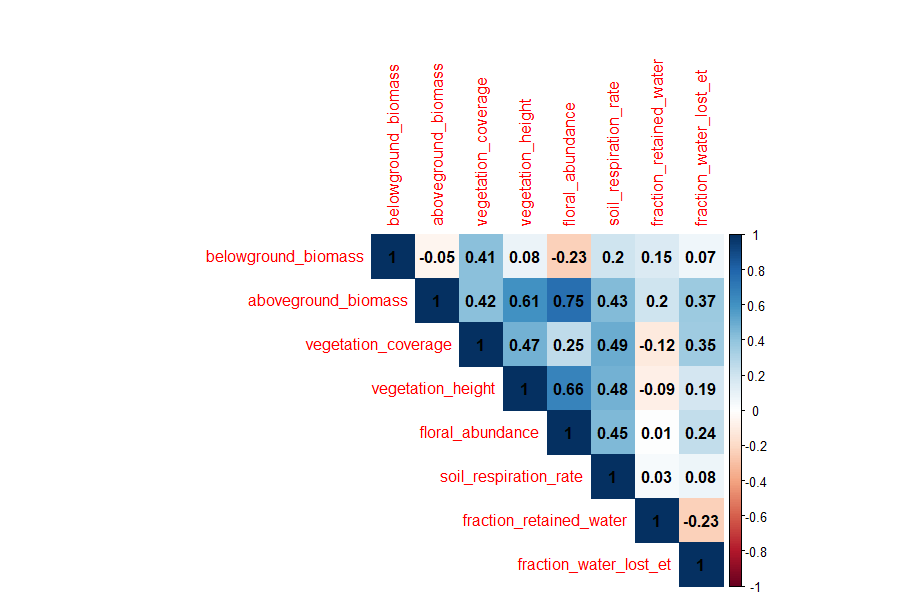


**S4 Fig. Correlation matrix for the eight indicator variables of ecosystem functions assessed on mesocosm grasslands.** We mimicked restored urban road verge grasslands in a mesocosms experiment in climate chambers of the TUMmesa ecotron facility. The red color indicates negative, while blue indicates positive correlations. A correlation value higher than |0.7| indicates strong collinearity between pairs of functions (Dormann et al. 2013).

**Literature cited**

Dormann CF, Elith J, Bacher S, Buchmann C, Carl G, Carré G et al. Collinearity: a review of methods to deal with it and a simulation study evaluating their performance. Ecography 2013;36(1):27–46
