# Supplementary material for: Low precipitation due to climate change consistently reduces multifunctionality of urban grasslands in mesocosms
Source: PLoS One. 2023 Feb 3;18(2):e0275044. doi: 10.1371/journal.pone.0275044 (PMC9897532; doi:10.1371/journal.pone.0275044)
Supplement: S5 Fig — (DOCX) [file pone.0275044.s007.docx]

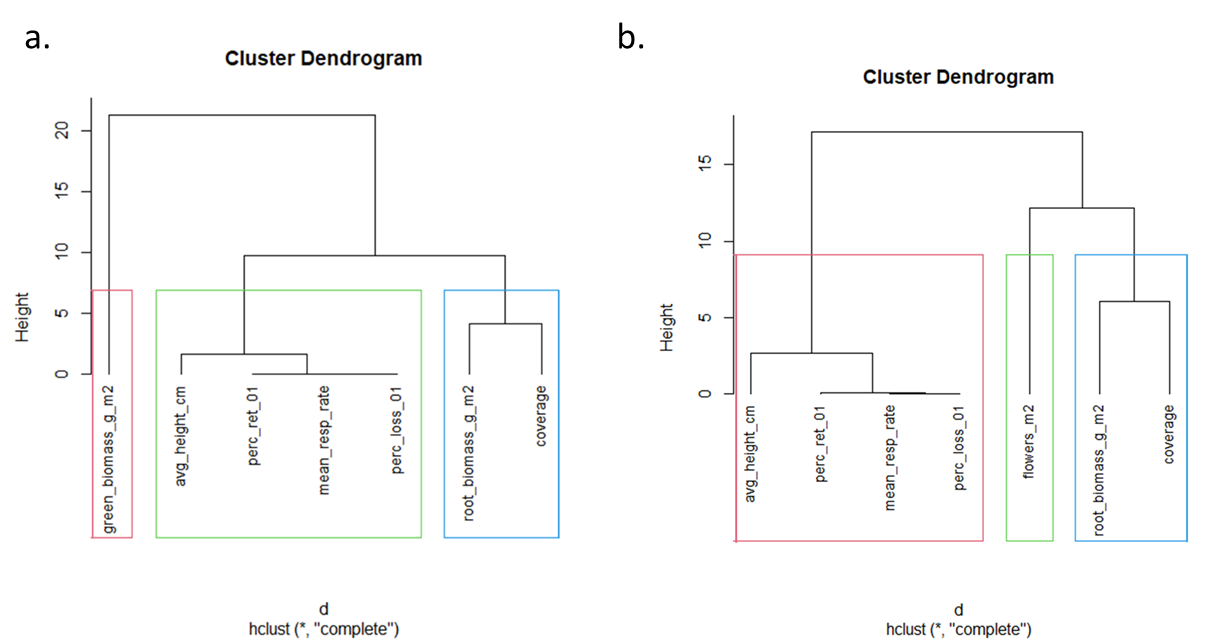


**S5 Fig. Clusters of seven indicator variables of ecosystem functions.** We measured indicators in experimental grasslands established in four *walk-in* chambers of TUMmesa ecotron subjected to climate change conditions (RCP scenarios). Given the high correlation between aboveground biomass and floral density, two clustering procedures were conducted including either aboveground biomass (a) or floral density (b). The indicator variables were used to assess mesocosm urban grasslands multifunctionality in response to climate change and functional composition. The clustering (via the elbow method) was used for downweighing closely related variables before calculating ecosystem multifunctionality.
