# Supplementary material for: Low precipitation due to climate change consistently reduces multifunctionality of urban grasslands in mesocosms
Source: PLoS One. 2023 Feb 3;18(2):e0275044. doi: 10.1371/journal.pone.0275044 (PMC9897532; doi:10.1371/journal.pone.0275044)
Supplement: S1 Table — (DOCX) [file pone.0275044.s008.docx]

**S1 Table. Species used in the mesocosm experiment to simulate urban grasslands**. Only native species and regionally produced seed material were used. Selection criteria for the forbs were their ability to adapt to ruderal environments like road verges, the provisioning of resources for pollinators, and diversity in functional traits related to plant performance, e.g., start of flowering, flower shape and color, specific leaf area, and maximum height. The selected native grass species are frequently observed in urban areas of Bavaria (Germany). * Legumes.

| **Species** | **Family** | **Functional type** |
| --- | --- | --- |
| *Daucus carota* L. | Apiaceae | Forbs |
| *Pastinaca sativa* L. | Apiaceae |  |
| *Achillea millefolium* L. | Asteraceae |  |
| *Centaurea jacea* L. | Asteraceae |  |
| *Centaurea scabiosa* L. | Asteraceae |  |
| *Cichorium intybus* L. | Asteraceae |  |
| *Crepis biennis* Lapeyr. | Asteraceae |  |
| *Cyanus segetum* Hill | Asteraceae |  |
| *Inula salicina* L. | Asteraceae |  |
| *Inula hirta* L. | Asteracee |  |
| *Echium vulgare* L. | Boraginaceae |  |
| *Berteroa incana* (L.) DC. | Brassicaceae |  |
| *Campanula rapunculoides* L. | Campanulaceae |  |
| *Scabiosa columbaria* L. | Caprifoliaceae |  |
| *Silene noctiflora* L. | Caryophyllaceae |  |
| *Anthyllis vulneraria* L.* | Fabaceae |  |
| *Lathyrus pratensis* L.* | Fabaceae |  |
| *Lotus corniculatus* L.* | Fabaceae |  |
| *Medicago falcata* L.* | Fabaceae |  |
| *Trifolium medium* L.* | Fabaceae |  |
| *Origanum vulgare* L. | Lamiaceae |  |
| *Salvia pratensis* L. | Lamiaceae |  |
| *Thymus pulegioides* L. | Lamiaceae |  |
| *Malva moschata* L. | Malvaceae |  |
| *Papaver rhoeas* L. | Papaveraceae |  |
| *Consolida regalis* Gray | Ranunculaceae |  |
| *Dactylis glomerata* L. | Poaceae | Grasses |
| *Festuca rubra* L. | Poaceae |  |
| *Holcus lanatus* L. | Poaceae |  |
| *Lolium perenne* L. | Poaceae |  |
| *Poa pratensis* L. | Poaceae |  |

Names verified on The World Flora Online – WFO (2022)
