# Supplementary material for: Low precipitation due to climate change consistently reduces multifunctionality of urban grasslands in mesocosms
Source: PLoS One. 2023 Feb 3;18(2):e0275044. doi: 10.1371/journal.pone.0275044 (PMC9897532; doi:10.1371/journal.pone.0275044)
Supplement: S2 Table — (DOCX) [file pone.0275044.s009.docx]

**S2 Table. Composition of each experimental grassland community with the sown proportion of the two functional types 'grasses' and 'forbs.'** Grassland communities are named as follows: grass-only (F0, 0% forbs), even-composed (F50, 50% forbs), forb-dominated (F75, 75% forbs), and forb-only communities (F100, 100% forbs). The final weight of seeds of each species in each mixture was determined using the formula of Burton et al. (2006), considering the desired percentage of the species, a final density of seed in the mixture of 4400 seeds/m^2^, the effective area of sowing in the experimental units (0.214 m^2^), and mass of the seeds per gram of each of the species. In each mixture, the evenness within each functional type was maintained at its maximum.

| **FT** | **Species** | **F0** | **F50** | **F75** | **F100** |
| --- | --- | --- | --- | --- | --- |
| Forbs | *Daucus carota* L. | 0 | 1.92 | 2.88 | 3.84 |
|  | *Pastinaca sativa* L. | 0 | 1.92 | 2.88 | 3.84 |
|  | *Achillea millefolium* L. | 0 | 1.92 | 2.88 | 3.84 |
|  | *Centaurea jacea* L. | 0 | 1.92 | 2.88 | 3.84 |
|  | *Centaurea scabiosa* L. | 0 | 1.92 | 2.88 | 3.84 |
|  | *Cichorium intybus* L. | 0 | 1.92 | 2.88 | 3.84 |
|  | *Crepis biennis* Lapeyr. | 0 | 1.92 | 2.88 | 3.84 |
|  | *Cyanus segetum* Hill | 0 | 1.92 | 2.88 | 3.84 |
|  | *Inula salicina* L. | 0 | 1.92 | 2.88 | 3.84 |
|  | *Inula hirta* L. | 0 | 1.92 | 2.88 | 3.84 |
|  | *Echium vulgare* L. | 0 | 1.92 | 2.88 | 3.84 |
|  | *Berteroa incana* (L.) DC. | 0 | 1.92 | 2.88 | 3.84 |
|  | *Campanula rapunculoides* L. | 0 | 1.92 | 2.88 | 3.84 |
|  | *Scabiosa columbaria* L. | 0 | 1.92 | 2.88 | 3.84 |
|  | *Silene noctiflora* L. | 0 | 1.92 | 2.88 | 3.84 |
|  | *Anthyllis vulneraria* L. | 0 | 1.92 | 2.88 | 3.84 |
|  | *Lathyrus pratensis* L. | 0 | 1.92 | 2.88 | 3.84 |
|  | *Lotus corniculatus* L. | 0 | 1.92 | 2.88 | 3.84 |
|  | *Medicago falcata* L. | 0 | 1.92 | 2.88 | 3.84 |
|  | *Trifolium medium* L. | 0 | 1.92 | 2.88 | 3.84 |
|  | *Origanum vulgare* L. | 0 | 1.92 | 2.88 | 3.84 |
|  | *Salvia pratensis* L. | 0 | 1.92 | 2.88 | 3.84 |
|  | *Thymus pulegioides* L. | 0 | 1.92 | 2.88 | 3.84 |
|  | *Malva moschata* L. | 0 | 1.92 | 2.88 | 3.84 |
|  | *Papaver rhoeas* L. | 0 | 1.92 | 2.88 | 3.84 |
|  | *Consolida regalis* Gray | 0 | 1.92 | 2.88 | 3.84 |
| Grasses | *Dactylis glomerata* L. | 20 | 10 | 5 | 0 |
|  | *Festuca rubra* L. | 20 | 10 | 5 | 0 |
|  | *Holcus lanatus* L. | 20 | 10 | 5 | 0 |
|  | *Lolium perenne* L. | 20 | 10 | 5 | 0 |
|  | *Poa pratensis* L. | 20 | 10 | 5 | 0 |

**Literature cited**

Burton, Carla M.; Burton, Philip J.; Hebda, Richard; Turner, Nancy J. (2006): Determining the Optimal Sowing Density for a Mixture of Native Plants Used to Revegetate Degraded Ecosystems. In *Restor Ecology* 14 (3), pp. 379–390. DOI: 10.1111/j.1526-100X.2006.00146.x
