# Supplementary material for: Low precipitation due to climate change consistently reduces multifunctionality of urban grasslands in mesocosms
Source: PLoS One. 2023 Feb 3;18(2):e0275044. doi: 10.1371/journal.pone.0275044 (PMC9897532; doi:10.1371/journal.pone.0275044)
Supplement: S3 Table — (DOCX) [file pone.0275044.s010.docx]

**S3 Table. Overview of indicator variables of urban grasslands functioning measured in the mesocosm experiment.**

| **Grassland function** | **Sign** | **Description** | **Role in urban grasslands** | **References** |
| --- | --- | --- | --- | --- |
| Aboveground biomass | **+** | Aboveground biomass harvested after 67 days of community establishment by clipping the vegetation 1 cm above soil level. The resulting material was oven-dried for 72 h at 70 °C and weighed | Plants constitute habitat and resources for fauna. Decreases in grassland productivity (less plant biomass) may translate in reduction of arthropods biomass. Carbon is sequestered in large extent by grasslands. The incorporation of resources into biomass increases the system metabolism and the use of available resources. It can be also consider as an indicator of productivity | Nagase and Dunnett (2012); Dusza et al. (2017) |
| Belowground biomass | **+** | Root biomass sampled by extracting two cores (height 15 cm, diameter 8 cm) per mesocosm after vegetation clipping (i.e., after 67 days of community establishment). The roots were washed, oven-dried at 60 °C for 72 h, and weighed | In grasslands, carbon storage occurs mainly in roots, which underlines their importance to replenish carbon stocks in the soil. Belowground growth of grassland species (root development) affects water infiltration and retention in the soil. Additionally, root growth of grasslands contributes to use of resources (N) that otherwise could leachate in water and increase urban water pollution | Bloor and Bardgett (2012); Bardgett et al. (2014);  Fry et al. (2018) |
| Flower production | **+** | The total number of flowers produced was determined by counting the floral units (sensu Baldock et al., 2015) per species at the 43rd day of grassland development; also mature flower buds were considered as produced flowers | Flowers are the resources for pollinating insects. Urban grasslands with a low management intensity regime produce abundant resources for pollinators. Aesthetics of diversified and flower-rich grasslands is highly appreciated by people living in cities | Hopwood (2008);  Blackmore and Goulson (2014);  Hoyle et al. (2018);  Tomitaka et al. (2021) |
| Vegetation height | **+** | Vegetation height was measured as the average of six height measurements (where the highest plant part touches a ruler) randomly performed within each mesocosm at the 43rd day of grassland development | Complexity or heterogeneity of vegetation is important in grassland habitats to improve the provision of habitat for insects. Surface temperature regulation is likewise a service of more complex grasslands. Greater water retention in the soil observed in grasslands with higher vegetation. Maximum vegetation height also correlates to productivity, while showing trade-offs in terms of drought resistance, water use efficiency, and soil fertility (although positively correlating to belowground characteristics) | Nagase and Dunnett (2012);  Bonthoux et al. (2014);  Lundholm (2015);  Norton et al. (2019);  Francoeur et al. (2021) |
| Vegetation cover | **+** | Cover of species (%) assessed on the total area of each experimental unit of 0.240 m^2;^ ; it was visually estimated at the 43rd day of grassland development | Increased vegetation cover can promote runoff reduction, that optimize the use of water for plant metabolism and likewise. Increased coverage also implies diminished leachates of nutrients to sewage water flows. Similarly, in small rainfall events, water is generally intercepted by the vegetation and subsequently evaporated, reducing the water runoff, and promoting temperature regulation and thermal comfort in cities | Armson et al. (2013);  Johnson et al. (2016) |
| Soil respiration | **+** | Soil respiration rate measured with an environmental gas Analyzer for CO_2_ (EGM-4. PP Systems) and a cylindrical PVC chamber (height = 150 mm, diameter = 100 mm) located on an area of bare soil inside the communities; four times in the 10th week of community development | Soil respiration is a key process in terrestrial carbon cycle. It positively correlates with temperature, precipitation, soil moisture and aboveground biomass. Precipitation is the primary factor controlling variation in respiration of grasslands. Higher precipitation might promote a higher CO_2_ release to the atmosphere under climate warming. Increases in soil respiration in urban grassland were shown to increase with increased management intensity (irrigation and fertilization) | Feng et al. (2018);  Meeran et al. (2021) |
| Water retention | **+** | Using a simulated heavy-rain event in each mesocosms, a watering-and- weighing protocol allowed for estimating water retention and loss by evapotranspiration. Each tray was weighed and then 15 L of water was evenly added over 4.5 min, representing a 71 mm of rain, corresponding to a heavy rain event recorded in Munich (May 2019). The trays were weighed again 1 h later; the difference between the first and second measurements was considered as captured water. After 24 h, each tray was weighed for the last time, and the difference between the third and second measurements was considered as water loss, mainly through evapotranspiration (ET). The same order of watering and weighing was followed to minimize time-related effects in the measurements. | Storm water regulation is supported by grasslands. Their attributes allow the infiltration of water aided by root penetration of the soil. The metabolism of grasslands allows for a rapid water cycling (due to plant growth, transpiration, etc.), a reduced charge to sewage systems and increased capture of water capacity after each storm event | MacIvor and Lundholm (2011);  Armson et al. (2013);  MacIvor et al. (2018) |
| Water loss by evapotranspiration | **+** |  | Storm water regulation involves also evapotranspiration (ET), as it helps reducing the amount of runoff water. Plants with high ET should improve retention performance of a grassland system. Temperature regulation in urban environments is aided by evapotranspiration, which reduces heat island effects | MacIvor and Lundholm (2011);  MacIvor et al. (2018);  Dusza et al. (2017) |

Indicator variables considered relevant for urban ecosystem functioning and the performance of ecosystem services were tested. The sign of the indicator variables was used to define needs of reflection of the functions when calculating multifunctionality (see Byrnes et al. 2014); (+) refers to high values of the variable deemed as desirable in urban grasslands, while (-) refers to undesired values of a given variable with potential impact on ecosystem services.

**Literature cited**

Armson, D.; Stringer, P.; Ennos, A. R. (2013): The effect of street trees and amenity grass on urban surface water runoff in Manchester, UK. In *Urban Forestry & Urban Greening* 12 (3), pp. 282–286. DOI: 10.1016/j.ufug.2013.04.001.

Baldock, K.C.R., Goddard, M.A., Hicks, D.M., Kunin, W.E., Mitschunas, N., Osgathorpe, L.M., Potts, S.G., Robertson, K.M., Scott, A.V., Stone, G.N., Vaughan, I.P., Memmott, J., 2015. Where is the UK's pollinator biodiversity? The importance of urban areas for flower-visiting insects. Proceedings of the Royal Society B: Biological Sciences 282 (1803), 20142849. doi:10.1098/rspb.2014.2849.

Bardgett, Richard D.; Mommer, Liesje; Vries, Franciska T. de (2014): Going underground: root traits as drivers of ecosystem processes. In *Trends in ecology & evolution*29 (12), pp. 692–699. DOI: 10.1016/j.tree.2014.10.006.

Blackmore, Lorna M.; Goulson, Dave (2014): Evaluating the effectiveness of wildflower seed mixes for boosting floral diversity and bumblebee and hoverfly abundance in urban areas. In *Insect Conserv Divers* 7 (5), pp. 480–484. DOI: 10.1111/icad.12071.

Bloor, Juliette M.G.; Bardgett, Richard D. (2012): Stability of above-ground and belowground processes to extreme drought in model grassland ecosystems: Interactions with plant species diversity and soil nitrogen availability. In *Perspectives in Plant Ecology, Evolution and Systematics* 14 (3), pp. 193–204. DOI: 10.1016/j.ppees.2011.12.001.

Bonthoux, Sébastien; Brun, Marion; Di Pietro, Francesca; Greulich, Sabine; Bouché-Pillon, Sabine (2014): How can wastelands promote biodiversity in cities? A review. In *Landscape and Urban Planning* 132, pp. 79–88. DOI: 10.1016/j.landurbplan.2014.08.010.

Chapin, F. Stuart; Matson, Pamela A.; Vitousek, Peter M. (Eds.) (2011): Principles of Terrestrial Ecosystem Ecology. New York, NY: Springer New York.

Dassonville, Nicolas; Vanderhoeven, Sonia; Vanparys, Valérie; Hayez, Mathieu; Gruber, Wolf; Meerts, Pierre (2008): Impacts of alien invasive plants on soil nutrients are correlated with initial site conditions in NW Europe. In *Oecologia* 157 (1), pp. 131–140.

Dusza, Yann; Barot, Sébastien; Kraepiel, Yvan; Lata, Jean-Christophe; Abbadie, Luc; Raynaud, Xavier (2017): Multifunctionality is affected by interactions between green roof plant species, substrate depth, and substrate type. In *Ecology and evolution* 7 (7), pp. 2357–2369. DOI: 10.1002/ece3.2691.

Feng, Jiguang; Wang, Jingsheng; Song, Yanjun; Zhu, Biao (2018): Patterns of soil respiration and its temperature sensitivity in grassland ecosystems across China. In *Biogeosciences*15 (17), pp. 5329–5341. DOI: 10.5194/bg-15-5329-2018.

Fischer, Leonie K.; Lippe, Moritz von der; Rillig, Matthias C.; Kowarik, Ingo (2013): Creating novel urban grasslands by reintroducing native species in wasteland vegetation. In *Biological Conservation* 159, pp. 119–126. DOI: 10.1016/j.biocon.2012.11.028.

Francoeur, Xavier W.; Dagenais, Danielle; Paquette, Alain; Dupras, Jérôme; Messier, Christian (2021): Complexifying the urban lawn improves heat mitigation and arthropod biodiversity. In *Urban Forestry & Urban Greening* 60, p. 127007. DOI: 10.1016/j.ufug.2021.127007.

Fry, Ellen L.; Savage, Joanna; Hall, Amy L.; Oakley, Simon; Pritchard, W. J.; Ostle, Nicholas J. et al. (2018): Soil multifunctionality and drought resistance are determined by plant structural traits in restoring grassland. In *Ecology* 99 (10), pp. 2260–2271. DOI: 10.1002/ecy.2437.

Grömping, Ulrike (2006): Relative Importance for Linear Regression in R: The Package relaimpo. In Journal of Statistical Software 17.

Herr, Cécile; Chapuis‐Lardy, Lydie; Dassonville, Nicolas; Vanderhoeven, Sonia; Meerts, Pierre (2007): Seasonal effect of the exotic invasive plant Solidago gigantea on soil pH and P fractions. In *J. Plant Nutr. Soil Sci.* 170 (6), pp. 729–738. DOI: 10.1002/jpln.200625190.

Hopwood, Jennifer L. (2008): The contribution of roadside grassland restorations to native bee conservation. In *Biological Conservation* 141 (10), pp. 2632–2640. DOI: 10.1016/j.biocon.2008.07.026.

Hoyle, Helen; Norton, Briony; Dunnett, Nigel; Richards, J. Paul; Russell, Jean M.; Warren, Philip (2018): Plant species or flower colour diversity? Identifying the drivers of public and invertebrate response to designed annual meadows. In *Landscape and Urban Planning* 180, pp. 103–113. DOI: 10.1016/j.landurbplan.2018.08.017.

Johnson, Catherine; Schweinhart, Shelbye; Buffam, Ishi (2016): Plant species richness enhances nitrogen retention in green roof plots. In *Ecological applications: a publication of the Ecological Society of America* 26 (7), pp. 2130–2144. DOI: 10.1890/15-1850.1.

Kayser, M.; Isselstein, J. (2005): Potassium cycling and losses in grassland systems: a review. In *Grass and forage science* 60 (3), pp. 213–224.

Li, Zhi-guo; Zhang, Guo-shi; Liu, Yi; Wan, Kai-yuan; Zhang, Run-hua; Chen, Fang (2013): Soil Nutrient Assessment for Urban Ecosystems in Hubei, China. In *PloS one*8 (9), e75856. DOI: 10.1371/journal.pone.0075856.

Lundholm, Jeremy T. (2015): Green roof plant species diversity improves ecosystem multifunctionality. In *J Appl Ecol* 52 (3), pp. 726–734. DOI: 10.1111/1365-2664.12425.

MacIvor, J. Scott; Lundholm, Jeremy (2011): Performance evaluation of native plants suited to extensive green roof conditions in a maritime climate. In *Ecological Engineering* 37 (3), pp. 407–417. DOI: 10.1016/j.ecoleng.2010.10.004.

MacIvor, J. Scott; Sookhan, Nicholas; Arnillas, Carlos A.; Bhatt, Anushree; Das, Shameek; Yasui, Simone-Louise E. et al. (2018): Manipulating plant phylogenetic diversity for green roof ecosystem service delivery. In *Evolutionary applications* 11 (10), pp. 2014–2024. DOI: 10.1111/eva.12703.

Maestre, Fernando T.; Quero, José L.; Gotelli, Nicholas J.; Escudero, Adrián; Ochoa, Victoria; Delgado-Baquerizo, Manuel et al. (2012): Plant species richness and ecosystem multifunctionality in global drylands. In Science (New York, N.Y.) 335 (6065), pp. 214–218. DOI: 10.1126/science.1215442.

Manning, Peter; van der Plas, Fons; Soliveres, Santiago; Allan, Eric; Maestre, Fernando T.; Mace, Georgina et al. (2018): Redefining ecosystem multifunctionality. In Nature ecology & evolution 2 (3), pp. 427–436. DOI: 10.1038/s41559-017-0461-7.

Meeran, Kathiravan; Ingrisch, Johannes; Reinthaler, David; Canarini, Alberto; Müller, Lena; Pötsch, Erich M. et al. (2021): Warming and elevated CO2 intensify drought and recovery responses of grassland carbon allocation to soil respiration. In *Global change biology* 27 (14), pp. 3230–3243. DOI: 10.1111/gcb.15628.

Mouillot, David; Villéger, Sébastien; Scherer-Lorenzen, Michael; Mason, Norman W. H. (2011): Functional structure of biological communities predicts ecosystem multifunctionality. In PloS one 6 (3), e17476. DOI: 10.1371/journal.pone.0017476.

Nagase, Ayako; Dunnett, Nigel (2012): Amount of water runoff from different vegetation types on extensive green roofs: Effects of plant species, diversity and plant structure. In *Landscape and Urban Planning* 104 (3-4), pp. 356–363. DOI: 10.1016/j.landurbplan.2011.11.001.

Norton, Briony A.; Bending, Gary D.; Clark, Rachel; Corstanje, Ron; Dunnett, Nigel; Evans, Karl L. et al. (2019): Urban meadows as an alternative to short mown grassland: effects of composition and height on biodiversity. In *Ecological applications : a publication of the Ecological Society of America* 29 (6), e01946. DOI: 10.1002/eap.1946.

Phillips, Benjamin B.; Bullock, James M.; Osborne, Juliet L.; Gaston, Kevin J. (2020): Ecosystem service provision by road verges. In J Appl Ecol 57 (3), pp. 488–501. DOI: 10.1111/1365-2664.13556.

Pouyat, Richard V.; Szlavecz, Katalin; Yesilonis, Ian D.; Groffman, Peter M.; Schwarz, Kirsten (2010): Chemical, Physical, and Biological Characteristics of Urban Soils. In Jacqueline Aitkenhead-Peterson, Astrid Volder (Eds.): Urban Ecosystem Ecology. Madison, WI, USA: American Society of Agronomy, Crop Science Society of America, Soil Science Society of America (Agronomy Monographs), pp. 119–152.

Tomitaka, Mahoro; Uchihara, Shoko; Goto, Akihito; Sasaki, Takehiro (2021): Species richness and flower color diversity determine aesthetic preferences of natural-park and urban-park visitors for plant communities. In *Environmental and Sustainability Indicators* 11, p. 100130. DOI: 10.1016/j.indic.2021.100130.
