# Supplementary material for: Low precipitation due to climate change consistently reduces multifunctionality of urban grasslands in mesocosms
Source: PLoS One. 2023 Feb 3;18(2):e0275044. doi: 10.1371/journal.pone.0275044 (PMC9897532; doi:10.1371/journal.pone.0275044)
Supplement: S4 Table — (DOCX) [file pone.0275044.s011.docx]

**S4 Table. Summary output of best models selected for responses of single indicator variables of grassland functioning.** Only explanatory variables that were included in the final models are shown. Indicators were modeled as a response to climate change scenario (RCP; two levels: 2.6 and 8.5), precipitation (Precip; two levels: normal and reduced), forb proportion (F; four levels: F0, F50, F75, F100). All models were analyzed with (generalized) linear mixed-effects models ([G]LMM) and included the main effects, and selected interactions. ∆AIC_null_ indicate the difference in AIC between the best model and the null model.

|  | **Aboveground biomass** | | | **Belowground biomass** | | | **Floral density** | | | **Plant cover** | | |
| --- | --- | --- | --- | --- | --- | --- | --- | --- | --- | --- | --- | --- |
|  | Estimate | Std. Error | t stat | Estimate | Std. Error | t stat | Estimate | Std. Error | z stat | Estimate | Std. Error | t stat |
| Intercept | 241.160 | 35.990 | **6.70** | 166.052 | 10.673 | **15.56** | 3.431 | 0.188 | **18.21** | 87.281 | 2.151 | **40.57** |
| RCP 8.5 | 54.150 | 44.440 | 1.22 | 11.459 | 11.535 | 0.99 | 0.544 | 0.210 | **2.59** | 7.312 | 1.962 | **3.73** |
| Precipitation_Red_ | -67.130 | 17.550 | **-3.83** | -13.505 | 6.885 | **-1.96** | -0.424 | 0.100 | **-4.24** | -11.375 | 1.645 | **-6.92** |
| F50 | 101.470 | 24.810 | **4.09** | -40.553 | 9.736 | **-4.17** | 1.524 | 0.151 | **10.11** | 0.875 | 2.326 | 0.38 |
| F75 | 86.610 | 24.810 | **3.49** | -58.919 | 9.736 | **-6.05** | 1.657 | 0.150 | **11.02** | -5.938 | 2.326 | **-2.55** |
| F100 | 124.490 | 24.810 | **5.02** | -69.071 | 9.736 | **-7.09** | 1.888 | 0.151 | **12.54** | -4.812 | 2.326 | **-2.07** |
| RCP 8.5: Precip_Red_ |  |  |  |  |  |  |  |  |  |  |  |  |
| Prec_Red_:F50 |  |  |  |  |  |  |  |  |  |  |  |  |
| Prec_Red_:F75 |  |  |  |  |  |  |  |  |  |  |  |  |
| Prec_Red_:F100 |  |  |  |  |  |  |  |  |  |  |  |  |
| RCP 8.5:F50 |  |  |  |  |  |  |  |  |  |  |  |  |
| RCP 8.5:F75 |  |  |  |  |  |  |  |  |  |  |  |  |
| RCP 8.5:F100 |  |  |  |  |  |  |  |  |  |  |  |  |
| **Marg./ Cond. R^2^** | 0.39 / | 0.54 |  | 0.48 / | 0.54 |  | 0.76 / | 0.79 |  | 0.55 / | 0.57 |  |
| AIC | 744.9 |  |  | 621.9 |  |  | 702.5 |  |  | 621.9 |  |  |
| **∆AIC_null_** | 24.47 |  |  | 34.42 |  |  | 62.77 |  |  | 145.86 |  |  |
| **S4 Table.** (continue) |  |  |  |  |  |  |  |  |  |  |  |  |
|  |  |  |  |  |  |  |  |  |  |  |  |  |
|  | **Plant height** | | | **Soil respiration** | | | **Water retention** | | | **Water loss (ET)** | | |
|  | Estimate | Std. Error | t stat | Estimate | Std. Error | t stat | Estimate | Std. Error | z stat | Estimate | Std. Error | z stat |
| Intercept | 14.711 | 1.899 | **7.75** | 0.080 | 0.006 | **14.38** | 0.283 | 0.251 | **1.13** | -3.794 | 0.115 | **-33.05** |
| RCP 8.5 | 9.995 | 2.370 | **4.22** | 0.043 | 0.005 | **7.86** | 0.517 | 0.318 | 1.63 | -0.296 | 0.118 | -2.50 |
| Precipitation_Red_ | 2.786 | 2.315 | 1.20 | -0.017 | 0.004 | **-4.34** | 1.043 | 0.193 | **5.39** | 0.672 | 0.131 | **-5.12** |
| F50 | 6.000 | 2.536 | 2.37 | -0.004 | 0.006 | -0.64 | 0.000 | 0.189 | 0.00 | 0.214 | 0.128 | 1.67 |
| F75 | 9.406 | 2.536 | 3.71 | 0.005 | 0.006 | 0.97 | -0.010 | 0.189 | -0.55 | 0.004 | 0.133 | 0.03 |
| F100 | 7.625 | 2.536 | 3.01 | -0.006 | 0.006 | -1.05 | -0.006 | 0.189 | -0.34 | 0.138 | 0.130 | 1.06 |
| RCP 8.5: Precip_Red_ | -16.115 | 2.071 | **-7.78** |  |  |  | -0.768 | 0.271 | **-2.83** | 0.485 | 0.186 | **2.62** |
| Prec_Red_:F50 | -10.333 | 2.929 | **-3.53** |  |  |  |  |  |  |  |  |  |
| Prec_Red_:F75 | -10.229 | 2.929 | **-3.49** |  |  |  |  |  |  |  |  |  |
| Prec_Red_:F100 | -5.708 | 2.929 | -1.95 |  |  |  |  |  |  |  |  |  |
| RCP 8.5:F50 | 14.708 | 2.929 | **5.02** |  |  |  |  |  |  |  |  |  |
| RCP 8.5:F75 | 4.229 | 2.929 | 1.44 |  |  |  |  |  |  |  |  |  |
| RCP 8.5:F100 | 5.000 | 2.929 | 1.71 |  |  |  |  |  |  |  |  |  |
| **Marg./ Cond. R^2^** | 0.84 / | 0.84 |  | 0.68 / | 0.69 |  | 0.81 / | 1.17 |  | 0.36 / | 0.36 |  |
| AIC | 389.6 |  |  | -333.5 |  |  | -75.8 |  |  | -452.2 |  |  |
| **∆AIC_null_** | 92.19 |  |  | 22.62 |  |  | 11.48 |  |  | 11.87 |  |  |
