# Supplementary material for: Low precipitation due to climate change consistently reduces multifunctionality of urban grasslands in mesocosms
Source: PLoS One. 2023 Feb 3;18(2):e0275044. doi: 10.1371/journal.pone.0275044 (PMC9897532; doi:10.1371/journal.pone.0275044)
Supplement: S5 Table — (DOCX) [file pone.0275044.s012.docx]

**S5 Table. Multiple comparisons of forb proportion effects on single indicator variables of grassland functionality.**

| **A) Response: Aboveground biomass** | | |  |  |
| --- | --- | --- | --- | --- |
| **Contrast** | **Estimate** | **Std. error** | **Statistic** | **Adj. p-value** |
| F0 - F50 | -101.474 | 24.813 | -4.090 | 0.001 |
| F0 - F75 | -86.612 | 24.813 | -3.491 | 0.005 |
| F0 - F100 | -124.495 | 24.813 | -5.017 | <0.001 |
| F50 - F75 | 14.862 | 24.813 | 0.599 | 0.932 |
| F50 - F100 | -23.021 | 24.813 | -0.928 | 0.790 |
| F75 - F100 | -37.883 | 24.813 | -1.527 | 0.429 |
|  |  |  |  |  |
| **B) Response: Belowground biomass** | | |  |  |
| **Contrast** | **Estimate** | **Std. error** | **Statistic** | **Adj. p-value** |
| F0 - F50 | 40.553 | 9.736 | 4.165 | 0.001 |
| F0 - F75 | 58.919 | 9.736 | 6.052 | <0.001 |
| F0 - F100 | 69.071 | 9.736 | 7.094 | <0.001 |
| F50 - F75 | 18.366 | 9.736 | 1.886 | 0.246 |
| F50 - F100 | 28.519 | 9.736 | 2.929 | 0.025 |
| F75 - F100 | 10.153 | 9.736 | 1.043 | 0.725 |
|  |  |  |  |  |
| **C) Response: Floral density** | | |  |  |
| **Contrast** | **Estimate** | **Std. error** | **Statistic** | **Adj. p-value** |
| F0 - F50 | -1.524 | 0.151 | -10.111 | <0.001 |
| F0 - F75 | -1.657 | 0.150 | -11.016 | <0.001 |
| F0 - F100 | -1.888 | 0.151 | -12.535 | <0.001 |
| F50 - F75 | -0.133 | 0.135 | -0.983 | 0.760 |
| F50 - F100 | -0.364 | 0.135 | -2.690 | 0.047 |
| F75 - F100 | -0.231 | 0.135 | -1.707 | 0.331 |
|  |  |  |  |  |
| **D) Response: Plant cover** | |  |  |  |
| **Contrast** | **Estimate** | **Std. error** | **Statistic** | **Adj. p-value** |
| F0 - F50 | -0.875 | 2.326 | -0.376 | 0.982 |
| F0 - F75 | 5.938 | 2.326 | 2.553 | 0.063 |
| F0 - F100 | 4.813 | 2.326 | 2.069 | 0.176 |
| F50 - F75 | 6.813 | 2.326 | 2.929 | 0.025 |
| F50 - F100 | 5.688 | 2.326 | 2.445 | 0.080 |
| F75 - F100 | -1.125 | 2.326 | -0.484 | 0.962 |

Forb proportion (four levels: F0, F50, F75, F100) indicates the forb:grass ratio sown in the mesocosms. P-value adjustment conducted with the Tukey method. Contrasts were calculated with the package emmeans.
