# Supplementary material for: Low precipitation due to climate change consistently reduces multifunctionality of urban grasslands in mesocosms
Source: PLoS One. 2023 Feb 3;18(2):e0275044. doi: 10.1371/journal.pone.0275044 (PMC9897532; doi:10.1371/journal.pone.0275044)
Supplement: S6 Table — (DOCX) [file pone.0275044.s013.docx]

**S6 Table. Pairwise comparisons of forb proportion effects in interaction with climate-change on plant height of mesocosm grasslands.**

| **Response: Plant height** | |  |  |  |  |  |
| --- | --- | --- | --- | --- | --- | --- |
| **Interaction RCP scenario and forb proportion** | | | |  |  |  |
| **Contrast** | **RCP scenario** | **Estimate** | **Std. Error** | **df** | **t ratio** | **p-value** |
| F0 - F50 | RCP 2.6 | -0.833 | 2.071 | 49 | -0.402 | 0.978 |
| F0 - F75 | RCP 2.6 | -4.292 | 2.071 | 49 | -2.072 | 0.176 |
| F0 - F100 | RCP 2.6 | -4.771 | 2.071 | 49 | -2.304 | 0.111 |
| F50 - F75 | RCP 2.6 | -3.458 | 2.071 | 49 | -1.670 | 0.350 |
| F50 - F100 | RCP 2.6 | -3.937 | 2.071 | 49 | -1.901 | 0.241 |
| F75 - F100 | RCP 2.6 | -0.479 | 2.071 | 49 | -0.231 | 0.996 |
| F0 - F50 | RCP 8.5 | -15.542 | 2.071 | 49 | -7.505 | <0.00  10 |
| F0 - F75 | RCP 8.5 | -8.521 | 2.071 | 49 | -4.115 | 0.001 |
| F0 - F100 | RCP 8.5 | -9.771 | 2.071 | 49 | -4.718 | <0.001 |
| F50 - F75 | RCP 8.5 | 7.021 | 2.071 | 49 | 3.390 | 0.007 |
| F50 - F100 | RCP 8.5 | 5.771 | 2.071 | 49 | 2.787 | 0.037 |
| F75 - F100 | RCP 8.5 | -1.250 | 2.071 | 49 | -0.604 | 0.930 |
| **Interaction Precipitation and forb proportion** | | | |  |  |  |
| **Contrast** | **Precipitation** | **Estimate** | **Std. Error** | **df** | **t ratio** | **p-value** |
| F0 - F50 | reduced | -3.021 | 2.071 | 49 | -1.459 | 0.470 |
| F0 - F75 | reduced | -1.292 | 2.071 | 49 | -0.624 | 0.924 |
| F0 - F100 | reduced | -4.417 | 2.071 | 49 | -2.133 | 0.157 |
| F50 - F75 | reduced | 1.729 | 2.071 | 49 | 0.835 | 0.838 |
| F50 - F100 | reduced | -1.396 | 2.071 | 49 | -0.674 | 0.906 |
| F75 - F100 | reduced | -3.125 | 2.071 | 49 | -1.509 | 0.440 |
| F0 - F50 | normal | -13.354 | 2.071 | 49 | -6.449 | <0.001 |
| F0 - F75 | normal | -11.521 | 2.071 | 49 | -5.564 | <0.001 |
| F0 - F100 | normal | -10.125 | 2.071 | 49 | -4.889 | <0.001 |
| F50 - F75 | normal | 1.833 | 2.071 | 49 | 0.885 | 0.812 |
| F50 - F100 | normal | 3.229 | 2.071 | 49 | 1.559 | 0.411 |
| F75 - F100 | normal | 1.396 | 2.071 | 49 | 0.674 | 0.906 |

Forb proportion (four levels: F0, F50, F75, F100) indicates the forb:grass ratio sown in the mesocosms. Contrasts were calculated with the package emmeans.
