# Supplementary material for: Low precipitation due to climate change consistently reduces multifunctionality of urban grasslands in mesocosms
Source: PLoS One. 2023 Feb 3;18(2):e0275044. doi: 10.1371/journal.pone.0275044 (PMC9897532; doi:10.1371/journal.pone.0275044)
Supplement: S7 Table — (DOCX) [file pone.0275044.s014.docx]

**S7 Table. Output summary of best models for the multifunctionality of mesocosm grasslands.** Only explanatory variables that were included in the final models are shown. Multifunctionality modeled as a response to climate change scenario (RCP; two levels: 2.6 and 8.5), precipitation (Precip; two levels: normal and reduced), forb proportion (F; four levels: F0, F50, F75, F100). All models were analyzed with linear mixed-effects models (LMM) and included the main effects, and selected interactions. ∆AIC_null_ indicate the difference in AIC between the best model and the null model. Multifunctionality indices were calculated considering either aboveground biomass (AGB) or flower density with other six indicator variables of grassland functioning: belowground biomass, plant cover, plant height, soil respiration, water retention, and water loss.

| **A) Response** | **Averaged Multifunct AGB** | | |  |  |  |
| --- | --- | --- | --- | --- | --- | --- |
|  | **Estimate** | **Std. Error** | **t stat** | **Marg./ Cond. R^2^** | **AIC** | **∆AIC_null_** |
| Intercept | **0.565** | **0.035** | **16.28** | 0.55/0.70 | -107 | 28.96 |
| RCP 8.5 | 0.069 | 0.042 | 1.64 |  |  |  |
| Precip_Red_ | **-0.059** | **0.019** | **-3.07** |  |  |  |
| F50 | -0.031 | 0.027 | -1.14 |  |  |  |
| F75 | -0.032 | 0.027 | -1.19 |  |  |  |
| F100 | -0.010 | 0.027 | -0.36 |  |  |  |
| RCP 8.5: Precip_Red_ | **-0.056** | **0.027** | **-2.06** |  |  |  |
| RCP 8.5:F50 | **0.130** | **0.038** | **3.40** |  |  |  |
| RCP 8.5:F75 | 0.067 | 0.038 | 1.75 |  |  |  |
| RCP 8.5:F100 | 0.031 | 0.038 | 0.81 |  |  |  |
|  |  |  |  |  |  |  |
| **B) Response** | **Averaged Multifunct Flower Dens** | | |  |  |  |
|  | **Estimate** | **Std. Error** | **t stat** | **Marg./ Cond. R^2^** | **AIC** | **∆AIC_null_** |
| Intercept | **0.510** | **0.031** | **16.24** | 0.60/0.72 | -170 | 33.55 |
| RCP 8.5 | 0.075 | 0.044 | 1.68 |  |  |  |
| Precip_Red_ | **-0.059** | **0.018** | **-3.20** |  |  |  |
| F50 | -0.017 | 0.026 | -0.65 |  |  |  |
| F75 | -0.017 | 0.026 | -0.66 |  |  |  |
| F100 | 0.003 | 0.026 | 0.13 |  |  |  |
| RCP 8.5: Precip_Red_ | **-0.060** | **0.026** | **-2.31** |  |  |  |
| RCP 8.5:F50 | **0.120** | **0.037** | **3.29** |  |  |  |
| RCP 8.5:F75 | **0.075** | **0.037** | **2.05** |  |  |  |
| RCP 8.5:F100 | 0.044 | 0.037 | 1.21 |  |  |  |
|  |  |  |  |  |  |  |
| **C) Response** | **70% Threshold Multifunct AGB** | | |  |  |  |
|  | **Estimate** | **Std. Error** | **t stat** | **Marg./ Cond. R^2^** | **AIC** | **∆AIC_null_** |
| Intercept | **0.417** | **0.068** | **6.15** | 0.33/0.38 | -21 | 9.55 |
| RCP 8.5 | **0.185** | **0.071** | **2.60** |  |  |  |
| Precip_Red_ | **-0.174** | **0.045** | **-3.84** |  |  |  |
| F50 | 0.036 | 0.064 | 0.57 |  |  |  |
| F75 | -0.057 | 0.064 | -0.89 |  |  |  |
| F100 | -0.016 | 0.064 | -0.24 |  |  |  |
|  |  |  |  |  |  |  |
| **D) Response** | **70% Threshold Multifunct Flower Dens** | | |  |  |  |
|  | **Estimate** | **Std. Error** | **t stat** | **Marg./ Cond. R^2^** | **AIC** | **∆AIC_null_** |
| Intercept | **0.327** | **0.055** | **5.99** | 0.43/0.46 | -47 | 17.51 |
| RCP 8.5 | **0.292** | **0.062** | **4.68** |  |  |  |
| Precip_Red_ | -0.026 | 0.053 | -0.50 |  |  |  |
| F50 | -0.026 | 0.053 | -0.50 |  |  |  |
| F75 | -0.078 | 0.053 | -1.49 |  |  |  |
| F100 | 0.005 | 0.053 | 0.10 |  |  |  |
| RCP 8.5: Precip_Red_ | **-0.234** | **0.074** | **-3.15** |  |  |  |
